# Gastric Outlet Obstruction Due to Malposition of Replacement Gastrostomy Tube

**DOI:** 10.5811/cpcem.2019.7.43626

**Published:** 2019-09-30

**Authors:** Brent A. Becker, Travis C. Walker

**Affiliations:** Wellspan York Hospital, Department of Emergency Medicine, York, Pennsylvania

## Abstract

A 78-year old male presented to the emergency department after accidental dislodgement of his chronic gastrostomy tube. A replacement gastrostomy tube was passed easily through the existing stoma and flushed without difficulty. Confirmatory abdominal radiography demonstrated contrast in the proximal small bowel, but the patient subsequently developed epigastric pain and refractory vomiting. Computed tomography revealed the tip of the gastrostomy tube terminating in the pylorus or proximal duodenum. This case highlights gastric outlet obstruction complicating the replacement of a gastrostomy tube and the associated radiographic findings.

## CASE PRESENTATION

**Image 1:**
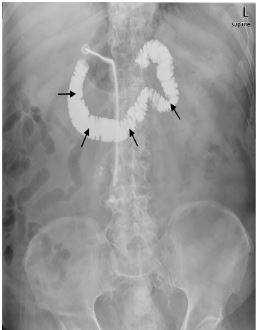
Contrast-enhanced abdominal radiography outlining the duodenum/proximal jejunum (arrows).

**Image 2:**
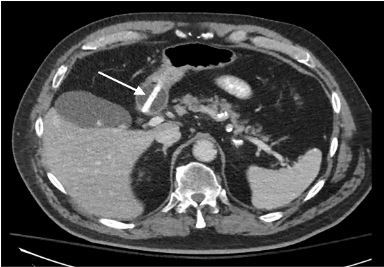
Abdominal computed tomography demonstrating distal portion of replacement gastrostomy tube (arrow) in the pylorus/proximal duodenum.

A 78-year-old male with a history of stroke presented to the emergency department after accidental dislodgement of his chronic gastrostomy tube approximately five hours prior. The patient offered no other complaints and denied abdominal pain, nausea, or vomiting. On examination, he appeared comfortable with unremarkable vital signs. His abdomen was non-tender and demonstrated a patent, mature gastrostomy stoma. A replacement gastrostomy tube was passed easily through the existing stoma and flushed without difficulty. Confirmatory abdominal radiography revealed contrast in the duodenum and proximal jejunum, but no portion of the stomach was outlined (Image 1). Shortly after, the patient developed epigastric pain, nausea, and refractory vomiting. Subsequent computed tomography revealed the tip of the gastrostomy tube terminating in the pylorus or proximal duodenum (Image 2). The balloon was deflated, and the tube was retracted several centimeters with complete resolution of symptoms. The patient was discharged home with no further complications on follow-up.

## DISCUSSION

Gastric outlet obstruction related to gastrostomy tubes is rare.^1^–^2^ Mechanical obstruction of the pylorus results in abdominal cramping and intermittent vomiting that resolve with tube repositioning.^1^,^3^ In the above case, the replacement gastrostomy tube was re-inserted directly into the gastric outlet; however, gastric outlet obstructions have been reported more in chronic indwelling catheters wherein dislodgement of the external bumper allows the tube to advance further into the stomach.^3^ Substitution of Foley catheters for true gastrostomy tubes confers a greater risk of gastric outlet obstruction, and this practice is discouraged.^1^,^4^

**CPC-EM Capsule**

What do we already know about this clinical entity?

*Dislodged gastrostomy tubes are commonly repositioned or replaced in the emergency department. Gastric outlet obstruction is a potential, albeit rare, complication*.

What is the major impact of the image(s)?

*Emergency physicians should be familiar with the appearance of gastric outlet obstruction due to gastrostomy tube malposition on contrast-enhanced plain radiography*.

How might this improve emergency medicine practice?

*Early identification of gastric outlet obstruction on plain radiography can prevent patient discomfort and the need for additional advanced imaging*.
